# In Vivo Delivery of Adenoviral Vector Containing Interleukin-17 Receptor A Reduces Cardiac Remodeling and Improves Myocardial Function in Viral Myocarditis Leading to Dilated Cardiomyopathy

**DOI:** 10.1371/journal.pone.0072158

**Published:** 2013-08-20

**Authors:** Yuquan Xie, Minghui Li, Xinggang Wang, Xian Zhang, Tianqing Peng, Yingzhen Yang, Yunzeng Zou, Junbo Ge, Haozhu Chen, Ruizhen Chen

**Affiliations:** 1 Key Laboratory of Viral Heart Diseases, Ministry of Public Health, Shanghai Institute of Cardiovascular Diseases, Zhongshan Hospital, Fudan University, Shanghai, China; 2 Department of Cardiology, Xinhua Hospital affiliated to Shanghai Jiaotong University, Shanghai, China; 3 Critical Illness Research, Lawson Health Research Institute, University of Western Ontario, London, Ontario, Canada; Albert Einstein College of Medicine, United States of America

## Abstract

Th17 cells have been implicated in the pathogenesis of myocarditis. Interleukin (IL)-17A produced by Th17 cells is dispensable for viral myocarditis but essential for the progression to dilated cardiomyopathy (DCM). This study investigated whether the adenoviral transfer of the IL-17 receptor A reduces myocardial remodeling and dysfunction in viral myocarditis leading to DCM. In a mouse model of Coxsackievirus B3 (CVB3)-induced chronic myocarditis, the delivery of the adenovirus-containing IL-17 receptor A (Ad-IL17RA:Fc) reduced IL-17A production and decreased the number of Th17 cells in the spleen and heart, leading to the down-regulation of systemic TNF-α and IL-6 production. Cardiac function improved significantly in the Ad-IL17R:Fc- compared with the Ad-null-treated mice 3 months after the first CVB3 infection. Ad-IL17R:Fc reduced the left ventricle dilation and decreased the mortality in viral myocarditis, leading to DCM (56% in the Ad-IL17R:Fc versus 76% in the Ad-null group). The protective effects of Ad-IL17R-Fc on remodeling correlated with the attenuation of myocardial collagen deposition and the reduction of fibroblasts in CVB3-infected hearts, which was accompanied by the down-regulation of A distintegrin and metalloprotease with thrombospondin type 1 motifs (ADAMTS-1), Matrix metalloproteinase-2(MMP-2), and collagen subtypes I and III in the heart. Moreover, in cultured cardiac fibroblasts, IL-17A induced the expression of ADAMTS-1, MMP-2, and collagen subtypes I and III and increased the proliferation of fibroblasts. We determined that the delivery of IL-17-RA:Fc reduces cardiac remodeling, improves function, and decreases mortality in viral myocarditis leading to DCM, possibly by suppressing fibrosis. Therefore, the adenoviral transfer of the IL-17 receptor A may represent an alternative therapy for chronic viral myocarditis and its progression to DCM.

## Introduction

Dilated cardiomyopathy (DCM) subsequent to myocarditis is a main cause of sudden death in youth and usually requires a heart transplant at the end stages of the disease [1], [2]. Henke et al. [3] reported that compared with normal mice infected with Coxsackievirus B3 (CVB3), CD4+ T cell knock-out mice infected with CVB3 demonstrated a lower virus titer in the myocardium, a lower mortality rate in the early stages of myocarditis and a more serious inflammatory outcome at the end stage, indicating that T cells mediate heart tissue injury in vivo. In addition, it has been reported that the adoptive transfer of myosin-specific CD4+ Th17 cells induces autoimmune myocarditis in normal mice [4], which demonstrates a crucial role for Th17 cells in the immune mechanism of myocarditis onset. Th17 cells are inflammatory cells first identified by Harrington [5], and the effective IL-17 molecule acts mainly on mesenchymal cells, such as fibroblasts, and induces the expression of cytokines, matrix metalloproteinase (MMP) and tissue inhibitor of metalloproteinase (TIMP) [6]. The role these cytokines play in the onset of viral myocarditis and experimental autoimmune myocarditis is controversial [7], [8]. It has been reported that the neutralization of IL-17A reduces serious autoimmune myocarditis [9]; however, we recently determined that in an acute viral myocarditis mouse model, the amount of Th17 cells and the expression of the related cytokines (IL-17A and IL-21) in the spleen increased significantly. In addition, the replication of CVB3 decreased significantly following IL-17-neutralizing antibody intervention [8].

IL-17A plays a vital role in the onset of many inflammatory diseases, mainly via inducing the expression of proinflammatory cytokines, such as IL-6, tumor necrosis factor, and a number of chemokines [10], [11]. Recent reports have shown that the neutralization of IL-17 using IL-17 receptor Ad:FC downregulates the expression of IL-6, tumor necrosis factor, etc., in colitis induced by trinitro-benzene-sulfonic acid [12], in atherosclerosis [13] and in concanavalin A-induced hepatitis [14]. The IL-17 receptor (IL-17R, mainly IL-17RA) is expressed in the heart tissue fibroblasts, and myocardial fibrosis is a key factor in the series of pathologic reactions that occur during the progression of myocarditis to DCM. Therefore, this study investigated the role of the adenovirus-containing IL-17 receptor A (Ad-IL17RA:Fc) in the progression of acute viral myocarditis to DCM, with emphasis on the occurrence of chronic fibrosis post-viral infection.

## Materials and Methods

### Animals and Virus

Pathogen-free, 4–6 week-old male BALB/c mice were purchased from the Joint Ventures Sipper BK Experimental Animal Company (Shanghai, China). The Animal Care and Use Committee of the Zhongshan Hospital, affiliated with Fudan University (No. 2009-11-203, Shanghai, China) approved the experimental protocols, which were performed in compliance with the “Guidelines for the Care and Use of Laboratory Animals”, published by the National Academy Press (NIH Publication No. 85–23, Revised 1996). The CVB3 (Nancy strain) was propagated in HeLa cells and titered as described previously [15].

### Cell Culture, Immunofluorescence Technology and Cardiac Fibroblast Proliferation Assay

Cardiac fibroblasts (CFs) were isolated from 3-day old Wistar rats by trypsinization, as described previously [16]. Briefly, the mouse hearts were minced and subjected to 15-min cycles of exposure to 0.125% trypsin (GIBCO, Grand Island, NY, USA) at 37°C. The trypsin-digested cells were collected by centrifugation at 300×*g* for 10 min. The cell pellet was resuspended in serum-containing medium, transferred to a Petri dish and incubated for 2.5 h in 5% CO_2_ at 37°C to allow the cells attach to the dish. The attached CFs were collected and plated at a cell density of 1×10^6^ cells/well onto six-well culture plates containing DMEM with 10% fetal calf serum. The next day,the medium was changed, and the confluent cells were passaged once. The culture contained more than 95% fibroblasts. Then, the CFs was treated with various concentrations of IL-17 (R&D Systems) or vehicle control. The IL-17-treated samples were subjected to immunofluorescence labeling using the primary antibody anti-rabbit DDR2 (1∶100, Santa Cruz Biotechnology) in combination with the nuclear dye DAPI (1∶100). The staining was performed in accordance with the instructions provided with the immunofluorescence kit. After the CFs were adhered, different concentrations of IL-17 were added to the wells, 5 ng/ml, 10 ng/ml, and 50 ng/ml, and DMEM containing 10% FCS was used as a negative control. The cells were incubated at 37°C in 5% CO_2_ for 48 h. Next, 10 µl of cell counting kit-8 (CCK-8) (Beyotime, China) was added to each well, and the plates were oscillated for 1 min on an oscillator and incubated at 37°C in 5% CO_2_ for 1.5 h. After incubation, the absorbance (A) was measured at 450 nm.

### Adenovirus Transfer and Groups

To examine the role of IL-17A in CFs, BALB/c mice were divided randomly into 3 groups and were injected *i.p.* with 100 µl of CVB3 diluted in DMEM on days 0 to induce the chronic viral myocarditis model. The treatment groups were as follows: (a) PBS group (*n = *20), the mice were injected *i.p* with 100 µl of PBS at same time of the period of chronic viral myocarditis; (b) Ad:IL-17R:Fc group (*n = *20), the mice were injected *i.p.* with Ad-IL-17AR:Fc at a concentration of 0.5×10^9^ PFU at age 14 days and with 1×10^9^ PFU of Ad-IL*-*17AR:Fc at 60 day of the period of chronic viral myocarditis; (c) Ad:null group (*n = *20), the mice were injected *i.p* with the Ad-null recombinant adenovirus 0.5×10^9^ PFU at age 14 days and 1×10^9^ PFU on day 60 of the period of chronic viral myocarditis. The vector encoding the soluble murine IL*-*17R:Fc fusion protein, which inhibits IL-17 activation specifically [17], was kindly provided by Dr. Jay K. Kolls (University of Pittsburgh, Children’s Hospital of Pittsburgh). The mice immunized with the Ad: null virus (Genechem Corporation, Shanghai, China) or PBS were used as the controls.

### Histopathology and Immunohistochemistry

Paraffin-embedded hearts were cut into 5 µm-thick tissue sections and were stained with hematoxylin &eosin (H&E) and picrosirius red to assess the myocardial injury and fibrosis. Briefly, the formalin-fixed tissue was stained with 0.1% picrosirius red F3BA dissolved in saturated picric acid, and the fields from the coded section were magnified using a Leica DMRD™ microscope (Leica, Germany). The total collagen content of 5 sections per sample was measured and expressed as an area fraction, which was calculated as the ratio of the thresholded chromogen area to the net myocardial area [18]. For the immunohistochemical analysis, paraffin-embedded sections were first deparaffinized and hydrated, microwave antigen retrieval was performed, endogenous peroxidase activity was blocked by incubation of the slides in 0.3% H_2_O_2_, and the non-specific binding sites were blocked using 4% BSA (Sigma). The slides were incubated with the primary antibody against collagen I and III (1∶200, Bioworld Technology, China) and the secondary antibody HRP-conjugated anti-rabbit (1∶500, Jackson ImmunoResearch laboratories, Inc, USA). Finally, the sections were developed in diaminobenzidine solution and counterstained with hematoxylin.

### Flow Cytometric Analysis

The splenocytes were isolated and suspended in RPMI 1640 containing 10% fetal bovine serum, and the RBCs were lysed by a 3 min incubation in ACK lysis buffer (TIANGEN). The cells were collected and resuspended at a density of 1.0×10^6^ cells/ml in a 24-well culture plate containing RPMI 1640 medium supplemented with 100 U/ml penicillin, 100 µg/ml streptomycin, and 10% FBS. Next, the cells were stimulated for 4 h with 50 ng/ml phorbol 12-myristate 13-acetate (PMA) and 1 µg/ml ionomycin (both from Sigma-Aldrich), and cytokine secretion was blocked using 10 µg/ml brefeldin A (Sigma-Aldrich) at 37°C and 5% CO_2._ The surface markers were stained using FITC-labeled anti-mouse CD4 antibody (RM4–5, BD Biosciences). After washing, fixing and permeabilizing in accordance with the manufacturer’s instructions (eBiosciences), the cells were stained intracellularly with PE-labeled anti-mouse IL-17A (TC11-18H10, BD Biosciences, San Jose, CA). Following incubation at 4°C for 30 min, the samples were washed in staining buffer, and the surface marker expression was measured by FACSCalibur flow cytometry. The data were analyzed using CellQuest software (BD Biosciences).

### Real-time PCR

The total RNA was isolated from each preparation using Trizol® reagent (Invitrogen) in accordance with the manufacturer’s instructions and converted into cDNA using M-MuLV reverse transcriptase (Fermentas). The cDNA was amplified using the SYBR green master mix (TaKaRa) and an ABI7500 thermocycler (Applied Biosystems). The primer pairs used were as follows: Col1α2: Forward TCGCTTCACCTACAGCACCCTTG and reverse GGTCTTGGTGGTTTTGTATTCG; Col3α1: Forward GTGGCTCTAATGGCATCAAAG and reverse ATGTGGTCCAACTGGTCCTCTG; a distintegrin and metalloprotease with thrombospondin type 1 motifs (ADAMTS-1): Forward ATCAGGACCAGGAAGCATAAGG and reverse CACCGAGAACAGGGTTAGAAGG; and GAPDH: Forward AGTATGATGACATCAAGAAGG and reverse ATGGTATTCAAGAGAGTAGGG. Relative expression levels were calculated according to the standard 2^−ΔΔCT^ method using GAPDH gene as endogenous control for normalization.

### Western Blotting

Samples containing 50 µg of protein were separated by 8% to 12% SDS-PAGE and were transferred to polyvinylidene difluoride membranes. The membranes were blocked with 2–5% BSA for 2 h at room temperature and incubated with anti-ADAMTS-1 antibody (1∶300, Santa Cruz Biotechnology), anti-MMP2 antibody (1∶1,000, Cell Signaling Technology), anti-TIMP1 antibody (1∶1,000, Cell Signaling Technology), anti-DDR2 antibody (1∶500, Santa Cruz Biotechnology), anti-IL-17A antibody (1∶1000, Cell Signaling Technology), anti-IL-17R antibody (1∶500, Santa Cruz Biotechnology) or anti-GAPDH antibody (1∶4000, Kangchen Biotech, China) at 4°C overnight. The membranes were then incubated with a secondary HRP-conjugated antibody (1∶4000, Jackson ImmunoResearch) for 1 h at room temperature. After incubation with the secondary antibody, the blots were developed using ECL (Thermo Scientific). The bands were analyzed using “Quantity One” software (Bio-Rad). The protein levels were expressed as the ratio of the band density relative to the GAPDH control band in the same sample.

### Cytokine Detection

The levels of IL-6 and TNF-α in serum, heart homogenates and cell culture supernatants were measured using the appropriate enzyme-linked immunosorbent assay (ELISA) kits (R&D systems) in accordance with the manufacturer’s instructions. The samples were measured in triplicate.

### Transthoracic Echocardiography

The animals were lightly anesthetized with inhalant isoflurane (2%) and imaged on a warm handling platform using a 30-MHz linear array transducer attached to a preclinical ultrasound system (Vevo 770, Visual Sonics Inc). M-mode and 2-D parasternal short-axis scans at the level of the papillary muscles were used to assess the changes in the left ventricle geometry and function to compare the left ventricle end-systolic inner diameter (LVIDS), left ventricle end-diastolic inner diameter (LVIDD), and fractional shortening (FS%). The left ventricle end-systolic volume (LV Vol;s) and end-diastolic volume (LV Vol;d) were acquired using the calculations 7/(2.4+LVIDS) × LVIDS^3^ and 7/(2.4+LVIDD) × LVIDD^3^, respectively.

### Statistics

The comparisons of the different treatment groups were performed using a one-way ANOVA. The data from the control and the AVMC groups at different time points were subjected to a two-way ANOVA. Survival was analyzed by the Kaplan-Meier method, and differences between the groups were tested using the log-rank test. The differences in the pathological scores were evaluated using the Mann-Whitney U-test. The comparison of the ratio of the heart weight to body weight was analyzed using the nonparametric Kruskal-Wallis test. The statistical package used was the SPSS 16.0 for Windows. The values are expressed as the mean ± SEM, with values of *P*<0.05 considered significant.

## Results

### Ad-IL-17AR:Fc Inhibits IL-17A, Reduces TH17 Cells and Down-regulates Systemic TNF-α and IL-6 in Viral Myocarditis Leading to DCM

**Figure 1:**
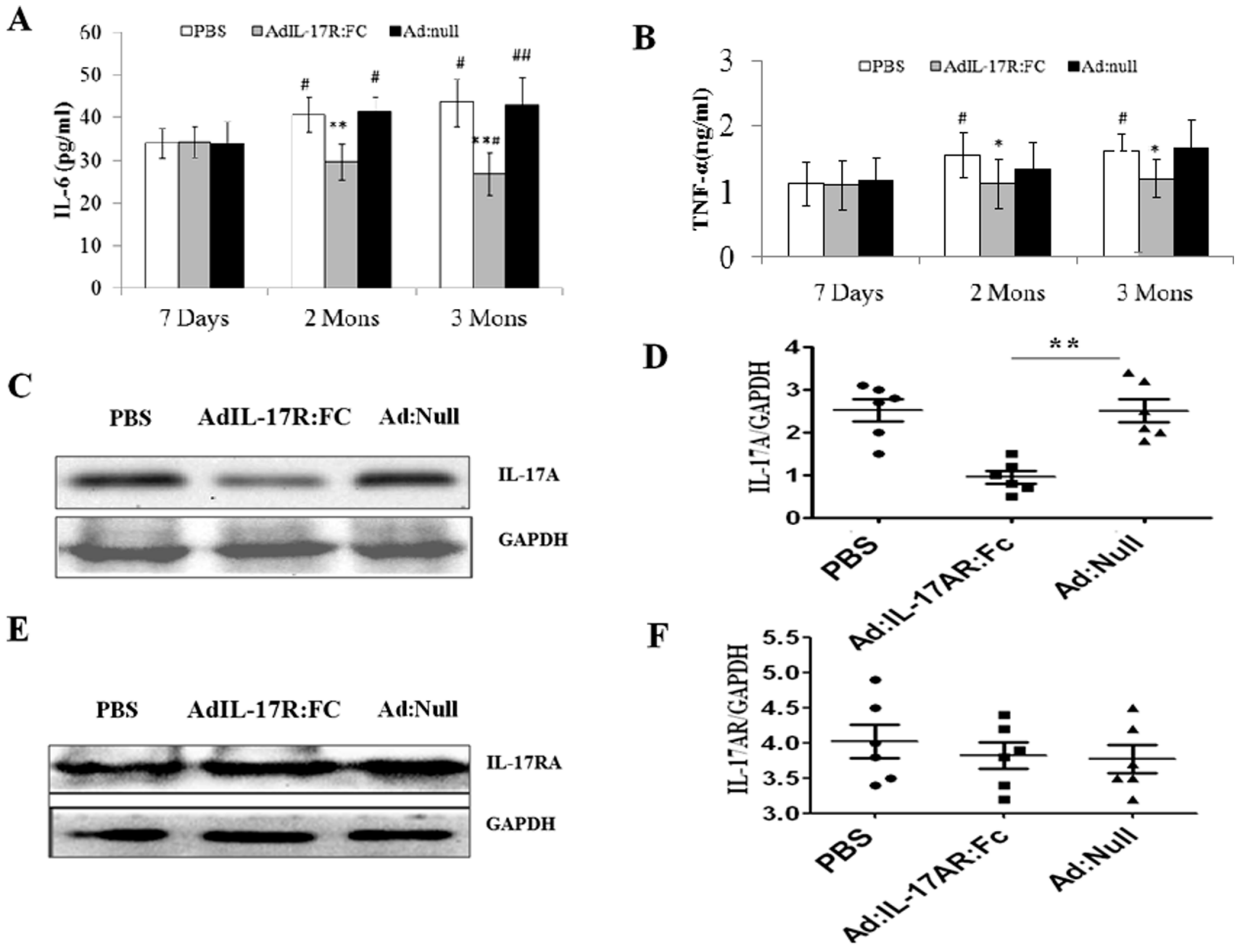
Ad-IL-17AR:Fc administration reduces IL-17A production in the blood and the heart. (*A*) Serum IL-6 concentrations at post-infection 7 days, 2mons and 3mons in PBS-, Ad-IL-17AR:Fc- and Ad:null-treated mice (n = 5) were measured using ELISA, and the data were analyzed by a 2-factor ANOVA. ^##^ *p*<0.01, ^#^ *p*<0.05 vs 7 Days group, **p<0.01, *p<0.05 vs control group, (*B*) Serum TNF-α concentrations at post-infection 7 days, 2mons and 3mons in PBS-, AdIL-17R:Fc- and Ad:null-treated mice (n = 5) were measured using ELISA, and the data were analyzed by a 2-factor ANOVA. ^#^ *p*<0.05 vs 7 Days group, *p<0.05 vs control group. The data represent the mean ± SEM for 6 mice per group. (*C*)The IL-17A protein extracted from the left ventricle of the mice in the three groups at post-infection day 90, was subjected to western blotting and was probed with the indicated antibodies (n = 3). (D)The IL-17A protein level is expressed as a ratio of the GAPDH level in the same sample; the data are expressed as the mean± SEM. *P<0.05 vs the control group. (*E*) The IL-17RA protein extracted from the left ventricle of the mice in the three groups at post-infection day 90, was subjected to western blotting and was probed with the indicated antibodies (n = 3). (*F*) The IL-17RA protein level is expressed as a ratio of the GAPDH level in the same sample; the data are expressed as the mean± SEM.

**Figure 2:**
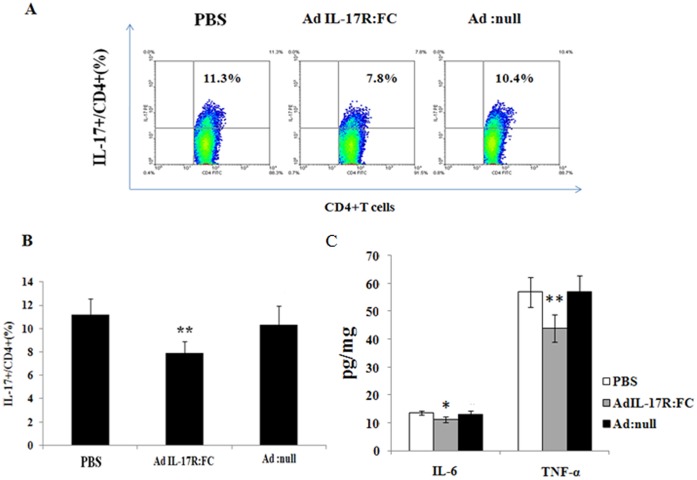
The number of Th17 cells in the spleen and cardiac tissue is decreased by Ad-IL-17AR:Fc. (*A*) Representative flow cytometry images of Th17 (CD4^+^ IL-17^+^) cells from each group gated on CD4^+^ T cells. Numbers in the upper right quadrants and lower right quadrants indicate the separate percentages of Th17 cells and CD4^+^ T cells, respectively. PE = phycoerythrin; FITC = fluorescein isothiocyanate. (*B*) The percentage of Th17 (CD4^+^ IL-17^+^) cells in each group was analyzed using CellQuest software. (*C*) The levels of IL-6 and TNF-α in the supernatants of heart homogenates detected using ELISA. The data represent the mean ± SEM for 6 mice per group. **P<0.01 vs control group, *p<0.05 vs control group.

Ad-IL-17AR:Fc has been used successfully to inhibit IL-17A via mediating IL-6 and TNF-α in a variety of mouse models. Firstly, It has been suggested that IL-17A induces TNF-α and IL-6 expression; therefore, we measured the TNF-α and IL-6 protein levels on the day 7, 60 and 90 post viral infection in the blood. The administration of Ad-IL-17AR:Fc consistently reduced the systemic TNF-α and IL-6 production on day 60 and 90 post-infection in viral myocarditis, especially on day 90 (Fig. 1A and B). Therefore, the results confirm that the delivery of Ad-IL-17AR:Fc reduces the expression of IL-17A on day 90 post-infection in chronic CVB3 myocarditis. To confirm the inhibitory effect of Ad-IL-17AR:Fc, we demonstrated that the myocardial IL-17A protein was reduced significantly in Ad-IL-17AR:Fc- compared with Ad-null-treated mice on day 90 post viral infection (Fig. 1C and D); however, Ad-IL-17AR:Fc did not alter the IL-17A receptor expression in the heart (Fig. 1E and F). To determine the effect of IL-17A inhibition by Ad-IL-17AR:Fc, we analyzed Th17 cells (CD4^+^ Th17^+^ T) in the spleen using flow cytometry. As shown in Fig.s 2A and 2B, the number of CD4^+^ Th17^+^ T cells is markedly increased in chronic CVB3 myocarditis. However, compared with the administration of Ad:null 90 days post viral infection, the administration of Ad-IL-17AR:Fc decreased the percentage of CD4^+^ Th17^+^ T cells. Additionally, the administration of Ad-IL-17AR:Fc consistently reduced the systemic TNF-α and IL-6 production in the supernatants of the heart homogenates on day 90 post-infection in viral myocarditis(Fig. 2C).

### Delivery of Ad-IL-17AR:Fc Improves Myocardial Function and Decreases the Mortality in Viral Myocarditis Leading to DCM

**Figure 3:**
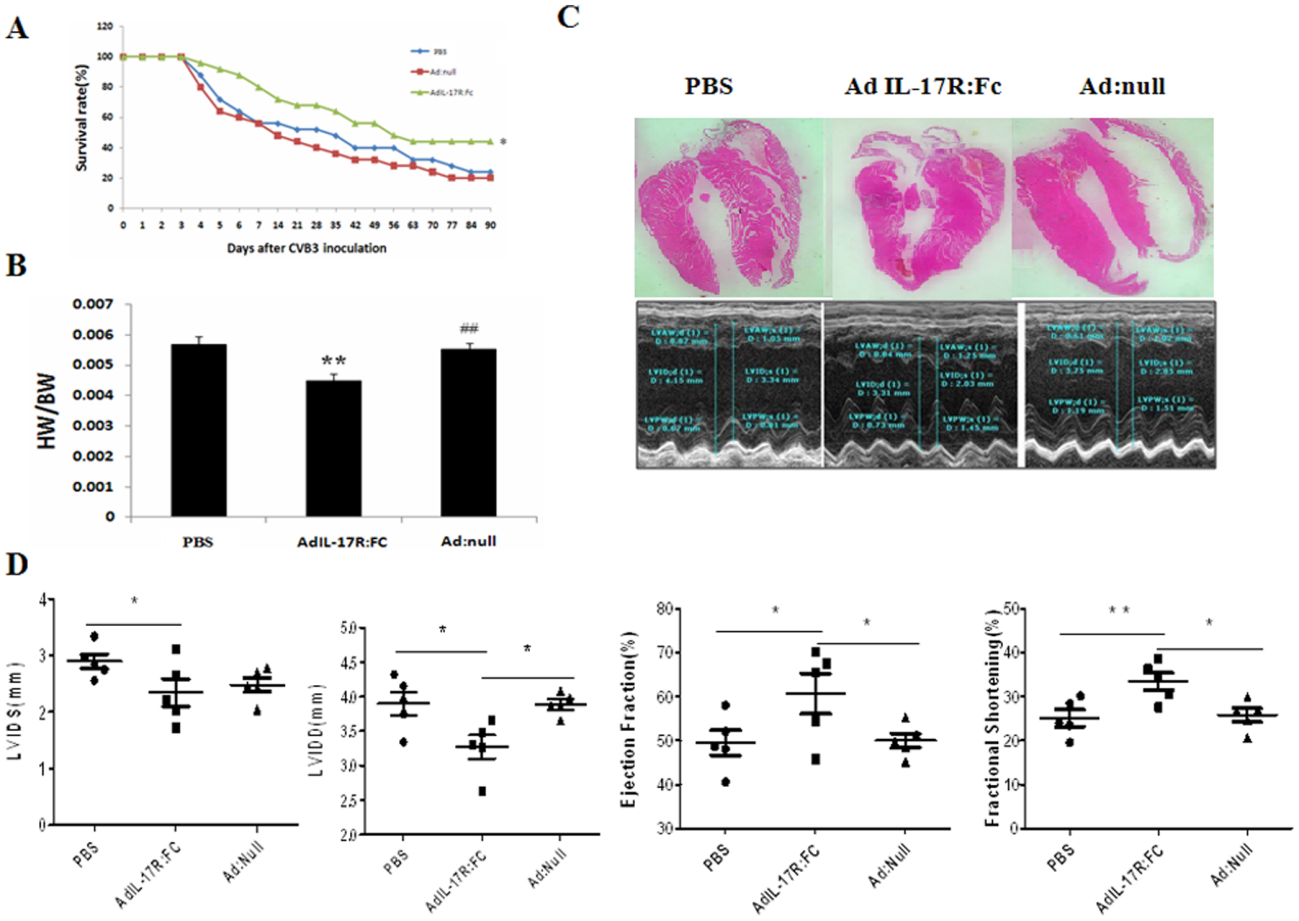
Cardiac remodeling reverses following treatment with Ad-IL-17AR:Fc in chronic viral myocarditis in mice. Groups of BALB/c mice were infused with Ad:null, Ad-IL-17AR:Fc or PBS. (*A*) Survival curves for mice after CVB3 infection. The mice in each group (n = 20) were monitored for up to 90 days after the intraperitoneal administration of CVB3. (*B*) Heart weight to body weight ratio (HW/BW) measured in five mice from the different groups (g/g). (*C*) Representative images of the heart from three mice (×50) and transthoracic M-mode tracings indicating the measurements. (*D*) The echocardiographic measurements are shown as the mean ± S.E.M. from five mice. LVIDS, Left ventricle internal dimension at end-systole; LVIDD, Left ventricle internal dimension at end-diastole; EF, Left ventricle ejection fraction; LVFS, Left ventricle fraction shortening. The values are expressed as the mean ± S.E.M. of five mice from each group. **p<0.01, *p<0.05 vs control.

To evaluate the effect of the IL-17A inhibition on cardiac function, echocardiography was performed 3 months after the first CVB3 injection. The systolic function index percent fractional shortening (FS) and ejection fraction (EF) demonstrated a significant improvement in cardiac function in Ad-IL-17AR: Fc- compared with Ad-null-treated mice (Fig. 3C and D). Similarly, Kaplan-Meier analyses demonstrated that the 90-day survival rate was much higher in the Ad-IL-17AR:Fc-treated group (44%) compared with the Ad-null-treated group (20%) (*p* = 0.035; Fig. 3A); however, the mortality in the PBS- and Ad-treated groups was not significantly different (*p*>0.05; Fig. 3A). The results demonstrate that the delivery of Ad-IL-17AR:Fc improves myocardial function and reduces mortality in viral myocarditis leading to DCM.

### Administration of Ad-IL-17AR:Fc Prevents Myocardial Remodeling in Viral Myocarditis Leading to DCM

The echocardiographic analysis performed 90 days after CVB3 infection revealed that the LV internal dimensions at the end diastole were significantly reduced in the Ad-IL-17AR:Fc-treated compared with the Ad-null-treated mice. Consequently, the LV end-diastolic volume was much smaller in the Ad-IL-17AR:Fc group than in the Ad-null group (Fig. 3C and D, Table S1), suggesting that inhibition of IL-17A prevents LV dilation in chronic myocarditis.

To substantiate the effect of Ad-IL-17AR:Fc on LV remodeling, we analyzed the LV cavity size. After H&E staining, the LV cavity size was measured at the papillary level (Fig. 3C). Consistent with the alteration observed in the echocardiography, the LV cavity size was significantly smaller in the Ad-IL-17AR:Fc-treated mice compared with the Ad-null-treated mice. In addition, Ad-IL-17AR:Fc decreased the ratio of heart weight over body weight in viral myocarditis leading to DCM (Fig. 3B).

**Figure 4:**
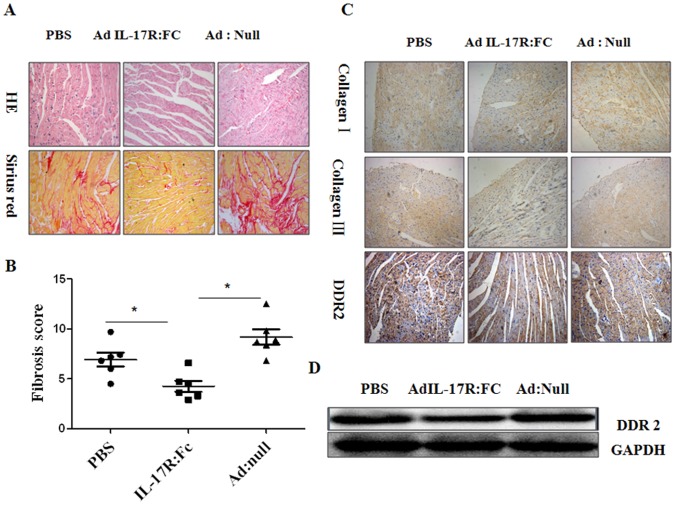
Reduction in myocardial total collagen content, collagen subtypes and proliferation of CFs following treatment with Ad-IL-17AR:Fc in chronic myocarditis. (*A*) H&E and picrosirius red staining to assess the myocardial injury and CFs in the different groups of mice. The original magnifications are ×200. (*B*) The total collagen content of 5 sections per sample was measured and expressed as the fibrosis score in the different groups of mice on day 90 after viral inoculation (n = 5). (*C*) Immunohistochemical analysis showing the change in Col I and Col III observed following neutralization of IL-17. The original magnifications are ×200. (*D*) Western blot showing DDR2 protein expression in the myocardium. The data are expressed as the mean ± SEM for 5 mice per group. *P<0.05 vs control group.

To examine further the effect of Ad-IL-17AR:Fc on cardiac remodeling in chronic myocarditis at the cellular level, we analyzed the total collagen deposition and the numbers of fibroblasts in the different groups of mice. Sirius red staining demonstrated a significant reduction in total collagen deposition in the Ad-IL-17AR:Fc-treated compared with the Ad-null-treated group (*p*<0.01) after CVB3 infection (Fig. 4A and B). Consistent with the reduction of collagen deposition, the levels of collagen subtypes I and III proteins were also decreased in the Ad-IL-17AR:Fc-treated compared with Ad-null-treated group (Fig. 4C). Furthermore, the immunohistochemical staining and western blot analysis for DDR2, a marker of fibroblasts, revealed the down-regulation of the DDR2 protein in the Ad-IL-17AR:Fc-treated group compared with the Ad:null- and PBS-treated groups (Fig. 4C and D). These results suggest that Ad-IL-17AR:Fc decreases the numbers of fibroblasts in myocardia after CVB3 infection.

### Ad-IL-17AR:Fc Reduces ADAMTS-1 and MMPs/TIMPs in Viral Myocarditis Leading to DCM

**Figure 5:**
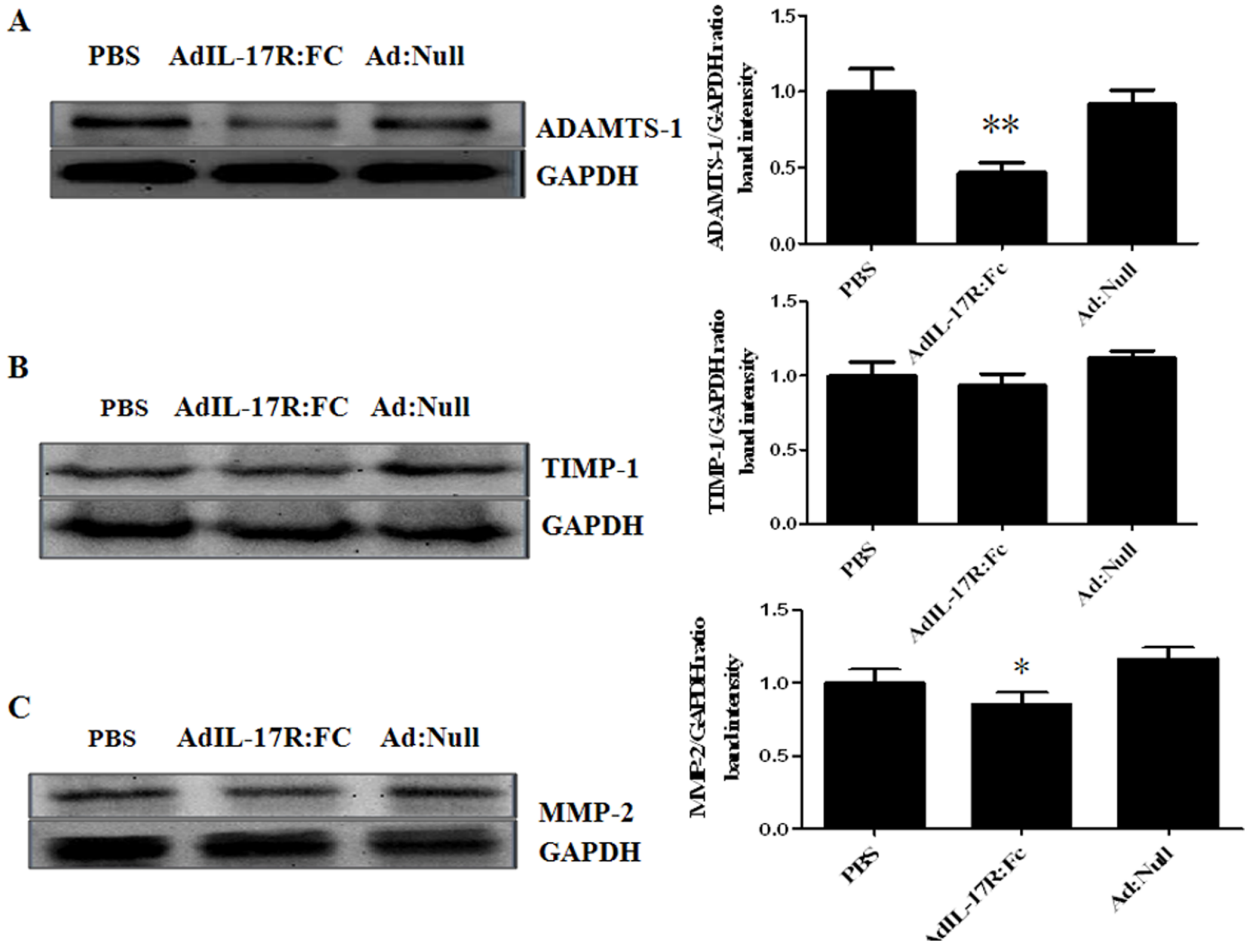
Degradation of CFs related to the MMPs/TIMPs expression following neutralization of IL-17. (*A*) Representative western blots showing the expression of the cardiac ADAMTS-1 protein in each group. (*B*) Representative western blots showing the expression of the cardiac TIMP-1 protein in each group. (*C*) Representative western blots showing the expression of the cardiac MMP-2 protein in each group. The expression of the cardiac ADAMTS-1 and MMP-2/TIMP-1 proteins in each group is expressed as a ratio of the GAPDH expression for the same sample. On day 90, 3 mice per group were analyzed. *P<0.05 vs control group.

The changes in ADAMTS-1 and MMPs/TIMPs are associated with ECM remodeling in the heart. To explore the relationship between IL-17A and ECM degradation from chronic viral myocarditis to DCM, we examined the expression of the ADAMTS-1 and MMPs/TIMPs proteins in the myocardium using western blotting. The expression levels of cardiac ADAMTS-1 and MMP-2 were lower in the Ad-IL-17AR:Fc-treated group than in the PBS- and Ad:null-treated groups (all *p*<0.05; Fig. 5A and C). However, the TIMP-1 levels did not differ between the Ad-IL-17AR:Fc-, PBS- and Ad:null-treated groups (*p*>0.05) (Fig. 5B). The results suggest that the effects of IL-17 inhibition on ECM remodeling correlate with the reduction in ADAMTS-1 and MMP-2 and the imbalance of MMP-2/TIMP-1 in chronic viral myocarditis.

### IL-17 induces MMPs/TIMP Protein Expression, Collagen Deposition and Cell Proliferation in Cultured Cardiac Fibroblasts

**Figure 6:**
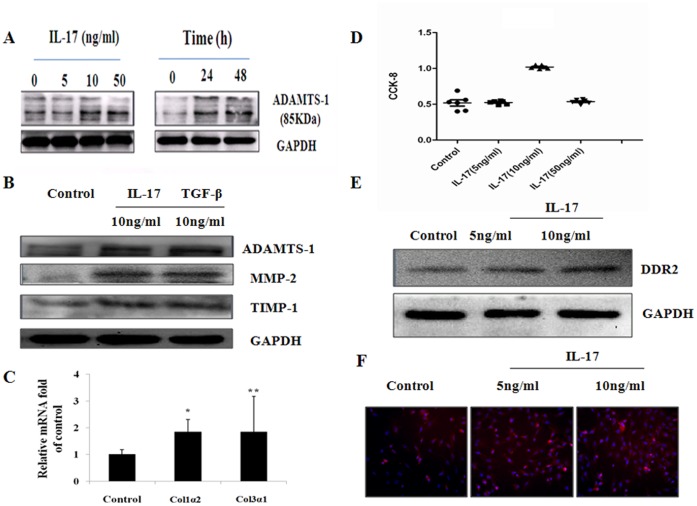
IL-17-stimulated MMPs/TIMPs protein expression, collagen deposition and proliferation in CFs. (*A*) Representative western blot for ADAMTS-1 protein levels in CFs treated with different doses (0, 10, and 50 ng/ml) of IL-17 for 24 h, and at 0, 24 and 48 h. (*B*) IL-17-mediated expression of ADAMTS-1 and MMP-2/TIMP-1 in the CFs treated with IL-17 (10 ng/ml) for 24 h, using TGF-β (10 ng/ml) as a control. (*C*) RT-PCR in CFs showing the IL-17-induced expression of COI1α2 and COI3α1 mRNAs. (*D*) The proliferation of CFs following the administration of different doses of IL-17 using cell counting kit-8 (CCK-8). (*E*) Western blot showing the expression of DDR2 at the different concentrations of IL-17. (*F*) Immunofluorescence in the cultured CFs showing the expression of the DDR2 protein in the 10 ng/ml IL-17, 5 ng/ml IL-17 and control groups (magnification ×200). The values in the IL-17-treated CFs were normalized to the values in the control cells (n = 5 per group). The data represent the mean ± SEM. *P<0.05 vs control group, **P<0.01 vs control group.

**Figure 7:**
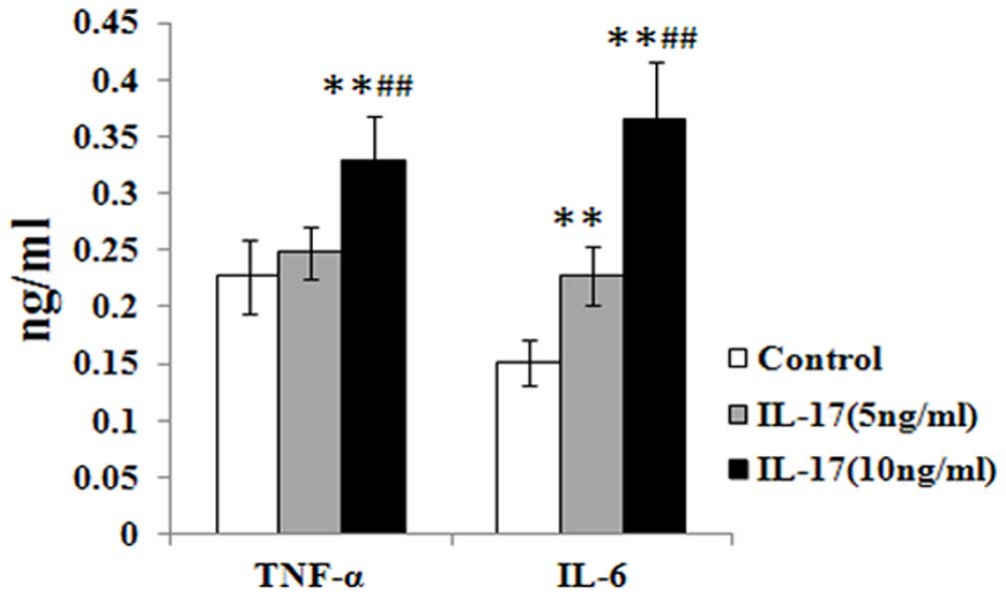
The levels of IL-6 and TNF-α in the supernatants of cardiac fibroblast cultures treated with IL-17. The IL-17 (10 ng/ml)-treated group produced higher levels of IL-6 and TNF-α cytokines than the control- and IL-17 (5 ng/ml)-treated groups. Moreover, IL-6 in the IL-17 (5 ng/ml)-treated group was highly expressed in the supernatants of the heart homogenates (ELISA). The values in the IL-17-treated CFs were normalized to the values in the control cells (n = 5 per group). The data represent the mean ± SEM. **P<0.01 vs control group, ^##^ *P*<0.01 vs control group.

To provide direct evidence demonstrating that IL-17 induces MMPs, ADAMTS-1, collagen deposition and proliferation, we stimulated cultured cardiac fibroblasts with IL-17. To investigate the optimal effect of IL-17 on ADAMTS-1 protein expression in cultured CFs, different doses of IL-17 (from 0 ng/ml to 50 ng/ml) were used. As shown in Fig. 6A, the 5 ng/ml dose of IL-17 increased ADAMTS-1 protein expression after 24 h of incubation, and 10 ng/ml of IL-17 had the largest effect on the expression of ADAMTS-1. Interestingly, the ADAMTS-1 protein expression was weaker at 50 ng/ml IL-17 than at 10 ng/ml IL-17. Therefore, in all subsequent experiments, 10 ng/ml IL-17 was used. IL-17 induced ADAMTS-1 expression in a time-dependent manner, with peak levels of protein observed at 24 h. We next investigated whether IL-17 also stimulates MMP-2 and TIMP-1 protein expression and collagen deposition using TGF-β (10 ng/ml) stimulation as a control. IL-17 and TGF-β stimulated ADAMTS-1 expression; however, the ADAMTS-1 expression induced by IL-17 was weaker than the TGF-β-induced ADAMTS-1 expression (Fig. 6B). Similarly, western blot analysis demonstrated that IL-17 induced MMP-2 and TIMP-1 protein expression in cultured CFs (Fig. 6B). Moreover**,** collagen deposition, which is attributed primarily to the increase in the COI1α2 and COI3α1 expression induced by IL-17, was demonstrated at the transcriptional level using RT-PCR in the CFs (Fig. 6C). To elucidate how IL-17 induces collagen deposition, we investigated the proliferation of CFs using cell counting kit-8 (CCK-8) and related the proliferation to the expression of DDR2, which is a specific marker of CFs. The studies indicated that the proliferation of CFs increased significantly with 10 ng/ml of IL-17 compared to both 5 ng/ml of IL-17 and to the control group (Fig 6D), whereas a sudden decrease in proliferation was observed at 50 ng/ml IL-17. However, western blotting (Fig. 6E) and immunofluorescence (Fig. 6F) analyses in the cultured CFs demonstrated that the expression of the DDR2 protein increased significantly following treatment with 10 ng/ml IL-17 compared with both 5 ng/ml IL-17 and with the control group. Finally, 10 ng/ml IL-17 induced higher levels of IL-6 and TNF-α cytokines than 5 ng/ml IL-17 in the cardiac fibroblast cultures. Moreover, IL-6 was highly expressed (demonstrated using ELISA) in the supernatants of the heart homogenates from the 5 ng/ml IL-17-treated mice (Fig. 7).

## Discussion

Using a chronic myocarditis model, this study demonstrated that the number of Th17 cells and the myocardium fibrotic scores increased significantly at 2 and 3 months post viral infection; the increase was more significant after 3 months than after 2 months (Fig. S1). The IL-6 and TNF-α levels decreased significantly on day 90 post viral infection, and myocardium fibrosis was ameliorated following blocking of IL-17A using an adenovirus and was accompanied by a significant decrease in viral myocarditis mouse mortality and a significant improvement in heart function.

A preliminary study [18] demonstrated that Th1 cells are the most important T-cell subgroup in the pathogenesis of CVB-induced myocarditis; the Th1 cells protected the myocardium from injury mainly by secreting the cytokine IFN-γ, which is associated with the negative regulation of RORγt, the transcription factor of the Th17 cell subtype [19], [20]. Our previous study [8] demonstrated that an IL-17-neutralizing antibody decreased the expression of IL-17A and IL-21, increased the IFN-γ yield, promoted the differentiation to the Th1 cell subgroup and inhibited virus replication effectively. Therefore, in the pathogenesis of viral myocarditis, Th17 cells facilitate virus proliferation mainly via suppressing the T cell differentiation into Th1 cells through IL-17. In addition, the proinflammatory function of Th17 cells plays an important role in the pathogenesis of various autoimmune diseases, such as autoimmune myocarditis [21]. A study [9] indicated that the extent of myocardium injury is ameliorated by the IL-17A neutralizing antibody in the autoimmune myocarditis model. However, Baldeviano et al. [7] demonstrated that during the onset of (myosin-induced) autoimmune myocarditis, IL-17A was not the most important factor in the early phase but played a significant role in the progression of myocarditis to DCM. Another study [22] demonstrated that in an epinephrine-induced heart failure model, IL-17 induced myocardium fibrosis via the RANKL/OPG and MMP/TIMP pathways, causing eventual heart failure.

To study the influence of IL-17 on the MMPs/TIMPs balance during viral infection, myocardium fibroblasts were stimulated in vitro using 10 ng/ml IL-17A, and the expression of MMP-2 and TIMP-1 increased significantly. The same outcome was observed in vivo in a viral myocarditis model, and furthermore, Ad-IL-17AR:Fc intervention decreased MMP-2 significantly without significantly changing TIMP-1 expression. The data indicate that over the course of viral myocarditis-induced myocardium fibrosis, the MMP/TIMP imbalance leads to the disruption of the post-transcriptional regulation of the myocardium collagen network. In addition, our in vivo study revealed that IL-17A upregulates the expression of ADAMTS-1, which is a member of the matrix metal protease family that can degrade type I collagen [23]. Our previous study of viral myocarditis using cDNA microarray analysis in the myocardium demonstrated that the expression of ADAMTS-1 increased significantly and the expression level correlated positively with the level of collagen deposition [24], [25]. These observations led to the hypothesis that the enhancement of Th17 cells and the effect of IL-17A on the extracellular matrix degradation are associated with the overexpression of ADAMTS-1 and the imbalance of MMPs/TIMPs. The observations in myocardium fibroblasts cultured in vitro demonstrated that IL-17A upregulates the expression of ADAMTS-1 in the myocardium in a time-dependent manner with the peak appearing after 24 hours. Compared with TGF-β, the IL-17A-induced ADAMTS-1 expression decreased significantly. Recent studies demonstrated that ADAMTS-1 binds VEGF and that the following VEGF-mediated signal transduction caused other effects, including antiangiogenesis [26]. These data indicate that the imbalance of ADAMTS-1 and MMP-2/TIMP-1 participates in the synthesis of the extracellular matrix and is involved in ventricular remodeling from the aspect of collagen degradation and angiogenesis.

In the course of tissue remodeling in many chronic autoimmune diseases, the synthesis and degradation of the extracellular matrix is influenced by the immunomicroenviroment of the focal inflammatory tissue [27]. The IFN-γ and IL-17A cytokines generated different focal immunomicroenviroments and regulated the MMPs/TIMPs balance and the extracellular matrix degradation in different ways [28]. Th17 cells may promote myocardial fibrosis through serial effectors, such as IL-6 and TNF-α. Studies in animal models of trinitro-benzene-sulfonic acid-induced colitis [12] and concanavalin A (Con A)-induced hepatitis [14] indicated that the blockade of IL-17A by IL-17R: FC is dependent primarily on the downregulation of IL-6 and TNF-α. Similarly, in the chronic viral myocarditis model, we determined that over the course of the progression of viral myocarditis to DCM, the lack of IL-17A stimulation leads to the significant downregulation of IL-6. IL-6 might facilitate myocardial fibrosis by regulating downstream IL-17A, promoting the progression of myocarditis into DCM. Another study [28] demonstrated that TNF-α over-expression in the early phase of left ventricular remodeling upregulated MMP activity and downregulated TIMP-1 expression and that IFN-γ protected the ECM from over-degradation by downregulating MMPs.

The data demonstrate that during the progression of viral myocarditis into DCM, IL-17A upregulates the expression of ADAMTS-1 and MMP-2 through different mechanisms, causes an imbalance in MMP2/TIMP-1 expression, increases the degradation of type I and type III collagen and eventually prompts the degradation of the extracellular matrix. In addition, the in vitro and in vivo experiments proved that IL-17 facilitates the proliferation of the myocardium fibroblasts. Therefore, the degradation of the extracellular matrix and the proliferation of myocardial fibroblasts together prompt the progression of myocarditis into DCM, leading to the increase in the extent of myocardium stiffness and eventually causing the disturbance of the heart systolic and diastolic functions.

## Supporting Information

Figure S1 **The fibrosis scores and myocardial dysfunction were significantly higher at 3 months compared to 2 months after repeated viral infection.** (*A*) The echocardiographic measurements are presented as the mean ± S.E.M. from ten mice. LVIDS, LV internal dimension at end-systole; LVIDD, LV internal dimension at end-diastole; EF, LV ejection fraction; LVFS, LV fraction shortening. (*B*) Representative flow cytometry images of Th17 (CD4^+^ IL-17^+^) cells, gated on CD4^+^ T cells, in each group. Numbers in the upper right quadrants and lower right quadrants indicate the percentages of Th17 cells and CD4^+^ T cells, respectively. PE = phycoerythrin; FITC = fluorescein isothiocyanate. (*C*) The percentage of Th17 (CD4^+^ IL-17^+^) cells in each group was analyzed using CellQuest software. (*D*) Picrosirius red staining to assess myocardial injury and CFs in the different groups of mice. Original magnifications are ×200. All values are expressed as the mean ± S.E.M. of six mice from each group. **p<0.01, *p<0.05 vs. control.
(TIF)

Table S1 **The echocardiographic measurements are shown in control and treatment groups.** The echocardiographic measurements are presented as the mean ± S.E.M. from five mice. LVIDS, LV internal dimension at end-systole; LVIDD, LV internal dimension at end-diastole; EF, LV ejection fraction; LVFS, LV fraction shortening.
(DOCX)
