# Supplementary figures and images for: In Vivo Delivery of Adenoviral Vector Containing Interleukin-17 Receptor A Reduces Cardiac Remodeling and Improves Myocardial Function in Viral Myocarditis Leading to Dilated Cardiomyopathy
Source: PLoS One. 2013 Aug 20;8(8):e72158. doi: 10.1371/journal.pone.0072158 (PMC3748008; doi:10.1371/journal.pone.0072158)

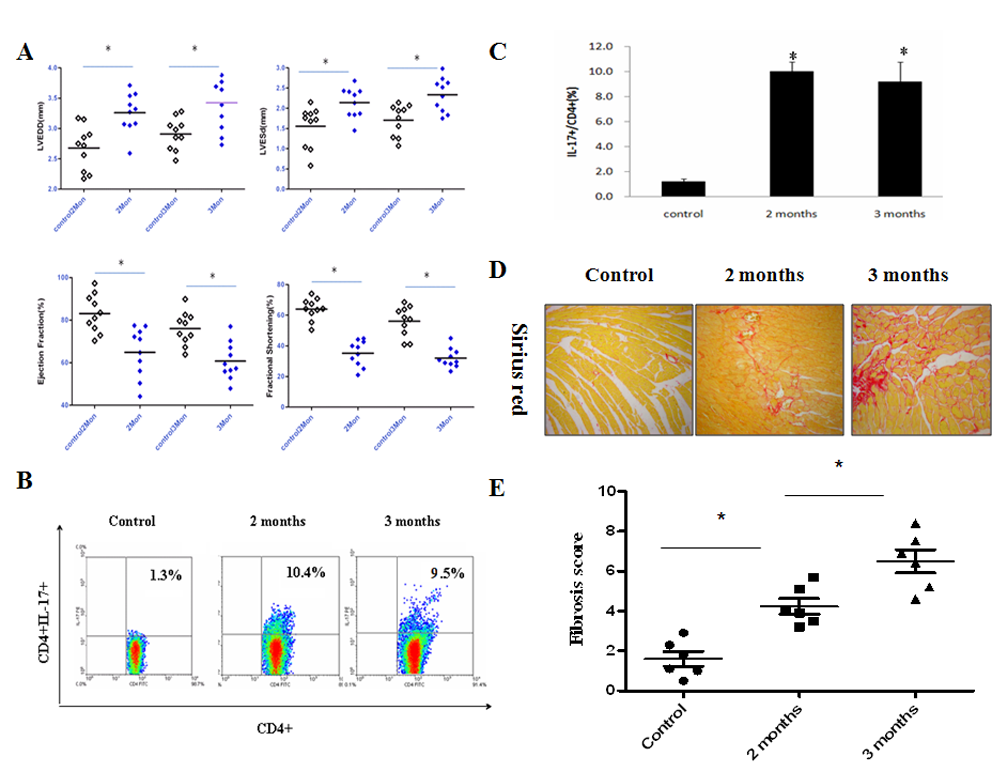

Supplement: Figure S1 — The fibrosis scores and myocardial dysfunction were significantly higher at 3 months compared to 2 months after repeated viral infection. (A) The echocardiographic measurements are presented as the mean ± S.E.M. from ten mice. LVIDS, LV internal dimension at end-systole; LVIDD, LV internal dimension at end-diastole; EF, LV ejection fraction; LVFS, LV fraction shortening. (B) Representative flow cytometry images of Th17 (CD4+ IL-17+) cells, gated on CD4+ T cells, in each group. Numbers in the upper right quadrants and lower right quadrants indicate the percentages of Th17 cells and CD4+ T cells, respectively. PE = phycoerythrin; FITC = fluorescein isothiocyanate. (C) The percentage of Th17 (CD4+ IL-17+) cells in each group was analyzed using CellQuest software. (D) Picrosirius red staining to assess myocardial injury and CFs in the different groups of mice. Original magnifications are ×200. All values are expressed as the mean ± S.E.M. of six mice from each group. **p<0.01, *p<0.05 vs. control. (TIF) [file pone.0072158.s001.tif]
