# Supplementary material for: In Vivo Delivery of Adenoviral Vector Containing Interleukin-17 Receptor A Reduces Cardiac Remodeling and Improves Myocardial Function in Viral Myocarditis Leading to Dilated Cardiomyopathy
Source: PLoS One. 2013 Aug 20;8(8):e72158. doi: 10.1371/journal.pone.0072158 (PMC3748008; doi:10.1371/journal.pone.0072158)
Supplement: Table S1 — The echocardiographic measurements are shown in control and treatment groups. The echocardiographic measurements are presented as the mean ± S.E.M. from five mice. LVIDS, LV internal dimension at end-systole; LVIDD, LV internal dimension at end-diastole; EF, LV ejection fraction; LVFS, LV fraction shortening. (DOCX) [file pone.0072158.s002.docx]

|  |  | 14days | 3 months |
| --- | --- | --- | --- |
| LVIDS (mm) | Control (n=5) | 1.41±0.28 | 1.73±0.37 |
|  | PBS (n=5) | 1.48±0.25 | 2.89±0.29 |
|  | Ad-IL-17AR:Fc (n=5) | 1.46±0.31 | 2.34±0.54 |
|  | Ad:null(n=5) | 1.44±0.27 | 2.48±0.29 |
| LVIDD (mm) | Control (n=5) | 1.98±0.21 | 2.92±0.26 |
|  | PBS (n=5) | 2.03±0.24 | 3.90±0.38 |
|  | Ad-IL-17AR:Fc (n=5) | 2.05±0.21 | 3.27±0.39 |
|  | Ad:null (n=5) | 2.07±0.25 | 3.87±0.16 |
| Ejection fraction (%) | Control (n=5) | 81.37±7.02 | 76.21±7.81 |
|  | PBS (n=5) | 75.28±7.32 | 49.50±6.36 |
|  | Ad-IL-17AR:Fc (n=5) | 77.05±6.98 | 60.71±10.28 |
|  | Ad:null (n=5) | 76.13±7.24 | 49.98±3.70 |
| Fractional shortening (%) | Control (n=5) | 60.65±5.43 | 56.03±9.87 |
|  | PBS (n=5) | 53.21±4.98 | 25.13±4.20 |
|  | Ad-IL-17AR:Fc (n=5) | 52.02±5.29 | 33.47±4.43 |
|  | Ad:null (n=5) | 51.67±5.06 | 25.81±3.42 |

Supplement Table S1 The echocardiographic measurements are shown in control and treatment groups.
